# A Novel Splice Variant of the Masculinizing Gene *Masc* with piRNA-Cleavage-Site Defect Functions in Female External Genital Development in the Silkworm, *Bombyx mori*

**DOI:** 10.3390/biom9080318

**Published:** 2019-07-30

**Authors:** Qin Zhao, Juan Li, Mao-Yu Wen, He Wang, Yao Wang, Kai-Xuan Wang, Qiu-Xing Wan, Xing-Fu Zha

**Affiliations:** 1 State Key Laboratory of Silkworm Genome Biology, Biological Science Research Center, Southwest University, Chongqing 400715, China; 2 School of Life Sciences, Southwest University, Chongqing 400715, China; 3 Chongqing Key Laboratory of Sericultural Science, Southwest University, Chongqing 400715, China; 4 Chongqing Engineering and Technology Research Center for Novel Silk Materials, Southwest University, Chongqing 400715, China

**Keywords:** silkworm, sex determination, alternative splicing, *Masc*, BmEXU, *Adb-B*

## Abstract

In the silkworm, the sex-determination primary signal *Fem* controls sex differentiation by specific binding of *Fem*-derived piRNA to the cleavage site in *Masc* mRNA, thus inhibiting *Masc* protein production in the female. In this study, we identified a novel splicing isoform of *Masc*, named *Masc-S*, which lacks the intact sequence of the cleavage site, encoding a C-terminal truncated protein. Results of RT-PCR showed that *Masc-S* was expressed in both sexes. Over-expression of *Masc-S* and *Masc* in female-specific cell lines showed that *Masc-S* could be translated against the *Fem*-piRNA cut. By RNA-protein pull-down, LC/MS/MS, and EMSA, we identified a protein BmEXU that specifically binds to an exclusive RNA sequence in *Masc* compared to *Masc-S*. Knockdown of *Masc-S* resulted in abnormal morphology in female external genital and increased expression of the Hox gene *Abd-B*, which similarly occurred by *Bmexu* RNAi. These results suggest that the splice variant *Masc-S* against *Fem*-piRNA plays an important role in female external genital development, of which function is opposite to that of full-length *Masc*. Our study provides new insights into the regulatory mechanism of sex determination in the silkworm.

## 1. Introduction

Sex determination and differentiation are fundamental processes that affect many aspects of an individual, such as external characteristics, physiology, behavior, etc. Insects exhibit a variety of sex-determining mechanisms, including male or female heterogamety and haplodiploidy [1]. The primary signal that starts sex determination is processed by a cascade of genes, ending with the conserved switch gene *doublesex* (*dsx*) that controls sexual differentiation [2]. The sex determination cascade in insects is conserved at the bottom but contains diverse primary signals at the top [3,4].

The silkworm, *Bombyx mori*, is a lepidopteran insect that has a ZZ/ZW sex chromosome system, in which females are heterogametic (ZW). A dominant feminizing factor *Fem* located in the W chromosome determines *B. mori* femaleness [5,6]. *Fem* RNA is transcribed from the W chromosome and produces female-specific PIWI-interacting RNA (piRNA) that targets mRNA of the protein-coding gene *Masculinizer* (*Masc*) [7]. Embryonic knockdown of the *Masc* gene resulted in feminization of male embryos, indicating that this gene encodes a masculinizing factor. Transgenic expression of the piRNA-resistant Masc Gene induced partial female-to-male sex reversal in the silkworm [8]. In females, *Fem*-piRNA formed a complex with Siwi, one of the *B. mori* PIWI proteins, and this complex specifically bound to the locus of the cleavage site within the ninth exon of the *Masc* gene to cleave *Masc* mRNA. In males, the *Masc* gene was translated into the protein product due to lack of *Fem*-piRNA [9]. Masc is a CCCH-tandem zinc finger (ZF) protein that contains 587 amino acid residues in the silkworm. Two cysteines at residues 301 and 304 are required for the masculinizing activity [10]. *Masc* mutant assays showed that deletion of the 210 C-terminal amino acids caused distinct defects in sexual differentiation, whereas two ZFs are dispensable for this function [11]. Homologs of *Masc* in other lepidopteran insects, including *Trilocha varians*, *Ostrinia furnacalis* and *Agrotis ipsilon*, were identified to function in masculinization [12,13,14]. Transfection of *Masc* cDNA into female-specific cells resulted in the production of male-type splicing of *B. mori dsx* gene (*Bmdsx*) [7]. In insects, sexually dimorphic abdominal morphology results from sex-specific gene regulation mediated by the Hox protein encoded by *Abdominal-B* (*Abd-B*) and products of the *dsx* gene [15]. *Abd-B* is also required to specify the posterior abdomen and the genitalia [16,17].

In this study, we cloned a novel splice variant of *Masc*, which lacks the intact sequence of the cleavage site for *Fem*-piRNA, and encodes a C-terminal truncated protein, to determine whether the splice variant is involved in female external genital development in the silkworm.

## 2. Materials and Methods

### 2.1. Silkworm Strains

The *Bombyx mori* non-diapausal strains (*D9L*) was provided from the Gene Resource Library of Domesticated Silkworm, Southwest University in China. The larvae were reared on fresh mulberry leaves at 25 ± 2 °C under a photoperiod of 12 h light/12 h dark with 75% relative humidity. Further, testis and ovary samples from the fifth instar larval stage were collected for isolation of total RNA and protein for different experimental analyses.

### 2.2. RT-PCR and Quantitative Real-Time RT-PCR (qPCR)

The total RNA was prepared using TRIzol^®^ (Invitrogen, Carlsbad, CA, USA) according to the manufacturer’s protocol. Subsequently, the cDNA was synthesized at 42 °C for 30 min by M-MLV reverse transcription (Promega, Madison, WI, USA). PCR was performed using specific primers designed to amplify the open reading frame (ORF) of genes and probable splice form of *Masc*, as well as to detect the sex-specific pattern of *Bmdsx* under different conditions. The corresponding primers for RT-PCR are shown in Supplementary Table S1. For qPCR, the reactions were run on an ABI7500 Real-Time PCR machine (Applied Biosystems, Foster City, CA, USA) with SYBR^®^ Premix Ex Taq™II (TaKaRa, Kusatsu, Japan). The eukaryotic translation initiation factor 4A (silkworm microarray probe ID sw22934) was used as the internal control [18]. We used the 2^−ΔΔCt^ method to analyze data.

### 2.3. Preparation of Polyclonal Antibody

For the preparation of polyclonal antibody against both Masc-S and Masc proteins, the common fragment of coding regions of Masc-S and Masc was amplified with cloning primers Masc-S-pro-f and Masc-S-pro-r (Supplementary Table S1). The PCR amplification was initiated by denaturation at 94 °C for 4 min, followed by 30 cycles of denaturation at 94 °C for 30 s, annealing at 54 °C for 30 s, and elongation at 72 °C for 45 s, followed by elongation at 72 °C for 10 min. PCR products were purified using the OMEGA gel extraction kit (Omega, Biel, Switzerland) and cloned into the pEASY-T1-simple vector (TransGen Biotech, Beijing, China). All clones were confirmed by sequencing. The fragment of Masc-S was released from the pEASY-T1-simple vector by digestion with BamH I and Hind III, and the fragment was ligated into the pET-28a prokaryotic expression vector (Novagen, Madison, WI, USA), which resulted in the fusion of a six-histidine tag to the amino terminus of Masc-S. Then, the recombinant vector pET-28a-Masc-S was transformed into Transetta (DE3) *Escherichia coli* competent cells. The bacteria were cultured in LB medium supplemented with 150 µg/mL Kanamycin in a shaker incubator at 16 °C or 37 °C at 230 rpm. When the optical density at 600 nm was 0.6, the bacteria were induced with 0.2 mM isopropyl β-D-1-thiogalactopyranoside (IPTG), and cells were further cultured for 12 h or 6 h at 200 rpm. The cells were collected by centrifugation and the cell pellet was resuspended in PBS. The bacteria were lysed by ultrasonication, centrifuged (12,000 rpm, 10 min) to separate the soluble and insoluble fractions and analyzed by sodium dodecyl sulfate-polyacrylamide gel electrophoresis (SDS-PAGE). Then, the Masc-S protein was purified by His-Trap column chromatography using the AKTA prime plus system (GE Healthcare, Chicago, IL, USA). Masc-S was eluted by elution buffer containing 500 mM imidazole, 500 mM NaCl and 20 mM Tris-HCl pH 7.5. Purified Masc-S was dissolved in phosphate-buffered saline (PBS) (pH 7.4), and it was verified by mass spectrometry (MS). The purified recombinant Masc-S protein was used to immunize New Zealand White rabbits to prepare a polyclonal antibody. The polyclonal antibody was purified with an antigen affinity chromatography column. Immunizing the rabbits and the antibody purification were finished in the commercial company (ZeHeng Biotech, China). The purified polyclonal antibody was used to detect the Masc-S protein in the silkworm.

### 2.4. Cell Culture and Transfection Assay

The *B. mori* cell lines BmE (referred to BmE-SWU1) and BmN (referred to BmN-SWU1), which were originally developed from embryonic and ovarian tissues [19,20], respectively, were obtained from State Key Laboratory of Silkworm Genome Biology. The cell lines were maintained at 27 °C in Grace medium (BmE) and IPL-41 medium (BmN) (GIBCO, Invitrogen) supplemented with 10% (*v*/*v*) fetal bovine serum (FBS) (Thermo Fisher Scientific, Waltham, MA, USA). BmE was used for electrophoretic mobility-shift assay (EMSA) of the BmEXU protein and the eBLE2 RNA sequence.

The full-length ORF of *Masc*, *Masc-S*, and *B. mori exuperantia* (*Bmexu*) with corresponding primers and different tag were generated. Specifically, *Masc* ORF and *Masc-S* ORF were amplified with Myc-tagged primers, and *Bmexu* ORF was amplified with His-tagged primers, as listed in Table S1. All sense and antisense primers contained BamHI and NotI sites, respectively. The target fragment was cloned into a BamHI and NotI-digested pSL1180 expression vector (conserved in our laboratory) to generate the recombinant expression vectors. The transfection was performed into the cell lines using the X-tremeGENE HP DNA transfection reagent (Roche, Basel, Switzerland) as described by the manufacturer’s instructions. Further, the pSL1180 basic transfection vector was used as the control. According to the previous study [8], we produced the Fem-piRNA-resistant MascR sequence by site-directed mutagenesis, and constructed the expression vector pSL1180-Myc-MascR.

### 2.5. Western Blotting Analysis

BmN cells transfected with the recombinant plasmids, pSL1180-Myc-Masc, pSL1180-Myc-Masc-S and pSL1180-Myc-MascR, respectively, were lysed in western and immunoprecipitation buffer (pH 7.5) containing 20 mmol/L Tris–HCl, 150 mmol/L NaCl, and 1% Triton X-100 (Beyotime, Shanghai, China). The lysates were mixed with loading buffer and boiled for 5 min. Proteins were separated by 12% (*w*/*v*) sodium dodecyl sulfate-polyacrylamide gel electrophoresis (SDS–PAGE) and electroblotted onto polyvinylidene fluoride (PVDF) membranes (Roche). The membranes were blocked overnight at 4 °C in 5% (*w*/*v*) skimmed milk, and directly incubated with the anti-Myc-tag antibody for 2 h at 37 °C and washed three times. Finally, the membranes were incubated with SuperSignal (R) West Pico chemiluminescent substrate (Thermo Fisher Scientific) and photographed using a ChemiScope chemiluminescent imaging system. The anti-tubulin antibody (Beyotime) were used to detect tubulin that was used as an internal control.

### 2.6. Subcellular Localization

As described above, silkworm cells were transfected with pSL1180-Myc-Masc-R, pSL1180-Myc-Masc-S and the basic pSL1180 vector, respectively. At 48 h post-transfection, the cells were incubated with a monoclonal anti-Myc antibody diluted 1:200 in PBS at 37 °C for 1 h. Finally, after six washes for 5 min each in PBS, the cells were incubated with Cy3-labeled goat anti-rabbit IgG (diluted 1:500, Promega) and DAPI (1:500; Thermo Fisher Scientific, Waltham, MA, USA) for 1 h. They were then washed six times with PBS, observed and imaged under a confocal microscope (FV3000, Olympus, Tokyo, Japan).

### 2.7. RNA-Protein Pull Down

First, the exclusive RNA sequence in *Masc* compared to *Masc-S*, we named as eBLE, was amplified with primers listed in Table S1. One of the two primers contained a T7 promoter site. Further, the complementary single RNA was synthesized using the RiboMAX™ Large Scale RNA Production Systems-T7 (Promega) and biotinylated using the Pierce™ RNA 3’ End Desthiobiotinylation Kit (Thermo Fisher Scientific). As a result, 5 nmol/L of RNA was prepared for incubation with magnetic beads (20–50 μL beads can capture 25–100 pmol RNA for reference). Following, 1.5 μg nucleoprotein extract from testis and ovary of the fifth instar larvae were incubated with the Pierce™ magnetic RNA-protein pull-down kit (Thermo Fisher Scientific). Subsequently, the beads with bound RNA-protein were washed by 1 × wash buffer three times and incubated with elution buffer at 37 °C for 30 min. After isolation by magnetic force, the supernatant fluid was collected and loaded on a 12% SDS-PAGE gel for analysis. Silver stain was performed to display the specific bands for sample collection. Each specific band, including peptide fragments, were removed and stored in deionized water at 4 °C to optimize spot destaining for subsequent in-gel tryptic digestion to recover peptide fragments for proteomics analysis by liquid chromatograph mass spectrometry (LC/MS/MS). All results were searched against UniProtKB Taxonomy-Bombyx mori (Silk moth) for peptide and protein identifications.

### 2.8. Electrophoretic Mobility-Shift Assay (EMSA)

EMSA was conducted to verify the combination between nucleic acid and protein according to the protocol of the chemiluminescent EMSA Kit (Pierce, Rockford, IL, USA). The 5’ terminus of the RNA oligonucleotide probes was labeled with biotin and synthesized in Tsingke, Beijing. The incubation of RNA probes and nucleoprotein extract proceeded on ice for 20 min. Subsequently, the mixture was loaded on a 5% (*w*/*v*) native polyacrylamide gel in TBE buffer (45 mM Tris-borate, 1 mM EDTA, pH 8.3) with 10 v/cm in an external ice bath. The probes were transferred electrophoretically to a proper nylon membrane with 380 mA for 20 min by trans-blot turbo transfer system (BioRad, Hercules, CA, USA). Further, the nylon membrane with samples was cross-linked under the following conditions: 254 nm UVC, 120 mJ/cm^2^, 45–60 s by UV-light cross-linker. The results were visualized using the SuperSignal™West Femto maximum sensitivity substrate (Thermo Fisher Scientific) and photographed by a Clinx ChemiScope 3400 Mini (Science Instrument, Shanghai, China).

### 2.9. RNA Interference (RNAi) Assay

Double-stranded RNAs (dsRNAs) was synthesized and transcribed using the RiboMAX™ Large-Scale RNA Production Systems-T7 (Promega). In addition, dsRNA of *Masc* and *Bmexu* was effectively generated from primers. Further, a negative control dsRNA was synthesized from the EGFP gene, and a T7 promoter site was added to all primers in Table S1. Subsequently, 50 μg and 60 μg dsRNA targeting *Bmexu* and *Masc*, respectively, were injected into each larva from the integument near the third pair of abdominal legs on day 1 of the fifth-instar using a glass capillary (laboratory-made from capillary glass tubing). Each treatment included 10 silkworms that were injected and three biological repeats were performed. After the 48-h or 72-h injection, the total RNA was extracted from the fat body and gonad for RT-PCR and qPCR analysis, and some test and control subjects were fed until moth development for observation and to record under the anatomical microscope.

## 3. Results

### 3.1. Identification of Novel Splice Isoform of Masc

**Figure 1:**
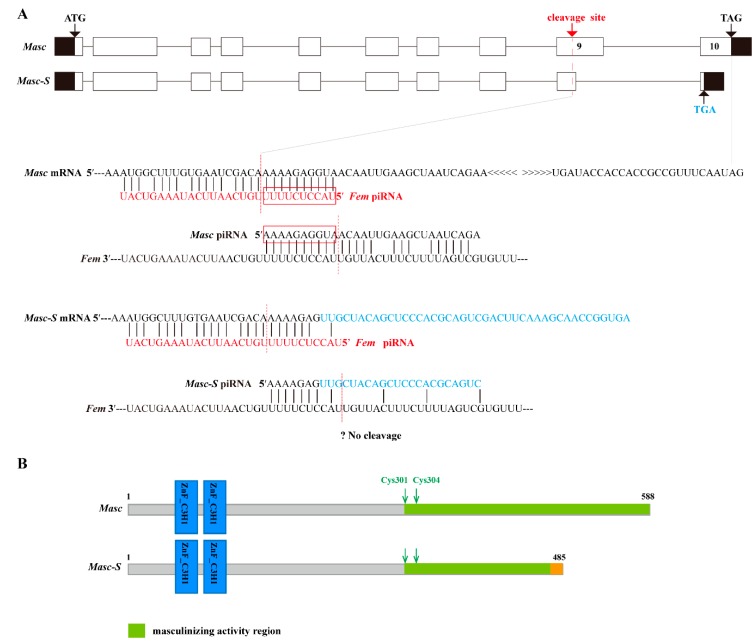
Schematic diagram for gene and protein domain structure of *Masc* and splice variant *Masc-S*. (**A**) The exonic structure of *Masc* and its splice isoform *Masc-S* was shown. The exons are represented by boxes and introns by a line. Coding and untranslated regions are indicated by white and black boxes, respectively. ATG: Starting codon, TAG/TGA: Stop codon. *Masc-S* lacked the sequence of nucleotides 1417–1603 of the *Masc* CDS, which is a part of exon 9. The red dotted line represents *Fem* piRNA cleavage site located in exon 9. For *Masc* mRNA, *Fem* piRNA and *Masc* piRNA both can participate in the ping-pong cycle of the piRNA amplification loop [7]. The red boxes represented the ping-pong signature. For *Masc-S* mRNA, the lacking region of the splice variant was closely adjacent to the *Fem* piRNA cleavage site (7 nt away), and the region of the cleavage site is different from *Masc* mRNA (shown as the sequence in blue). The putative *Masc-S* piRNA, if any, is unable to work. (**B**) Schematic diagram of the protein structure of *Masc* and *Masc-S*.

Based on the full-length CDS of the *Masc* gene, we designed specific primers and performed PCR. Results showed that there were two bands in the PCR product (Supplementary Figure S1). The bands were cloned and sequenced, demonstrating two sequences at 1767 and 1580 bp, respectively. The upper band was the same as the *Masc* CDS, and the lower lacked the sequence of nucleotides 1417–1603 of the *Masc* CDS. The shorter sequence was named as *Masc-short* (*Masc-S*) and submitted to GenBank (Acc. No. MK341694). Sequence analysis showed that the lacking sequence of *Masc-S* was located in the ninth exon of *Masc*, which contains a cleavage site of *Fem* piRNA, and the lacking region was closely adjacent to the cleavage site (Figure 1A). Further, *Masc-S* conformed to the splicing rule of GT/AG in the border of introns and exons, which suggested that *Masc-S* is a novel splicing isoform of *Masc*. Comparison of *Masc* and *Masc-S* protein indicated that the former contained two CCCH-type zinc finger domains, C-terminal masculinizing activity region, and the conservative site Cys-301, Cys-304. The latter differed in the C-terminal due to the lacking sequences, but the Cys-301, Cys-304 and shorter masculinizing activity region remained (Figure 1B).

### 3.2. Expression of Masc-S in the Silkworm and Cell Line

**Figure 2:**
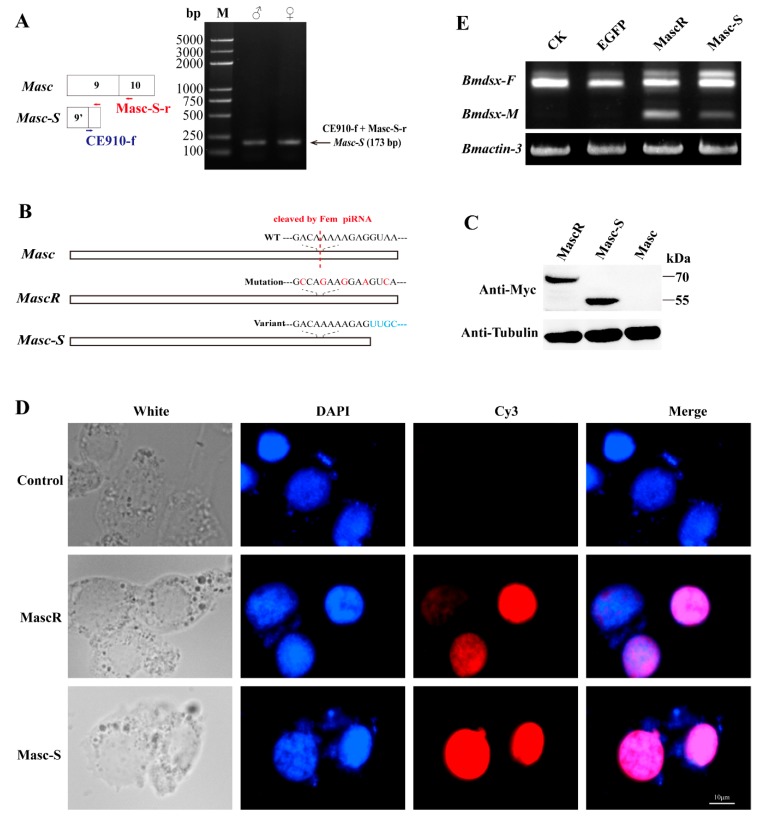
Expression of *Masc-S* in the silkworm and cell line. (**A**) RT-PCR for *Masc-S* in both sexes. The reverse primer Masc-S-r lies in exon 10 of Masc as well as Masc-S. The forward primer CE910-f, specific to *Masc-S*, overlaps the junction of exons 9’ and 10. PCR production by using the CE910-f and Masc-S-r primers was cloned and sequenced in order to confirm the sequence. (**B**,**C**) Over-expression of full-length *Masc*, splice variant *Masc-S* and piRNA-resistant *MascR* in the female-specific cell line BmN. MascR contains five nucleotide substitutions (red characters) from the wild-type (WT) sequence, which makes it resistant to cleavage by *Fem* piRNA [8]. The Myc-tagged *Masc*, *MascR* and *Masc-S* coding sequences were cloned into the pSL1180 vector, respectively. The recombinant expression vectors pSL1180-Myc-Masc, pSL1180-Myc-MascR and pSL1180-Myc-Masc-S, were transfected into the silkworm female-specific cell line BmN. Western blot was performed to detect Masc, MascR and Masc-S proteins with anti-Myc-tag antibody, with α-tubulin used for the internal control. (**D**) Subcellular location of MascR and Masc-S in the silkworm cell lines. DAPI-treated nuclei, red fluorescence for Cy3-treated MascR and Masc-S proteins, and the merged images are shown. (**E**) Effect of *Masc-S* over-expression on sex-specific alternative splicing of *Bmdsx*. Line CK represents the control, lines *EGFP*, *MascR* and *Masc-S* mean that psl1180-Myc-EGFP, psl1180-Myc-MascR and psl1180-Myc-Masc-S vectors were transfected into BmN, respectively. *Bmactin-3* was used for internal control. The band of male-specific splice type (*Bmdsx-M*) of *Bmdsx* was induced when *Masc-S* was over-expressed in BmN. However, expression level of *Bmdsx-M* induced by *Masc-S* is less than that by *MascR*.

The lacking region of *Masc-S* was close to the *Fem* piRNA cleavage site, possibly affecting the cleaving event. To verify the above analysis, we designed specific primers to amplify *Masc-S* specifically in each sex of the silkworm. RT-PCR showed that *Masc-S* was expressed in the ovary and testis of the silkworm (Figure 2A). Moreover, we constructed the recombinant vector pSL1180-Myc-Masc-S, which can be over-expressed in silkworm cell lines. Similarly, the recombinant vectors, pSL1180-Myc-Masc (wild type) and pSL1180-Myc-MascR (the mutant *Fem* piRNA cleavage site) were constructed (Figure 2B). All the above recombinant proteins were labeled with Myc tags and expressed in silkworm female-specific ovary cells BmN (containing W chromosome with *Fem* piRNA). Western blot was applied to detect the expression of the target protein using the anti-Myc-tag antibody, and there was no *Masc* protein expression in the silkworm ovary cell line. Conversely, expression of MascR and Masc-S showed protein hybridization signals located at 70 kDa and 55 kDa, respectively (Figure 2C). The results indicated that *Masc*, due to its piRNA binding cleavage site, could not form a protein in BmN, but the splicing variant *Masc-S* could resist the cleaving of *Fem* piRNA in the female-specific cells, resulting in a truncated protein. Furthermore, immunofluorescence analysis showed that the Masc-S protein was present in the nucleus, but not in the cytoplasm (Figure 2D), which cellular location is the same as the full-length MascR protein. Furthermore, we checked sex-specific alternative splicing of *Bmdsx.* Results showed that male-specific splice type (*Bmdsx-M*) of *Bmdsx* was induced when *Masc-S* was over-expressed in BmN, and that expression level of *Bmdsx-M* induced by *Masc-S* is less than that by *MascR*. These results indicated Masc-S retained partial masculinizing activity (Figure 2E).

**Figure 3:**
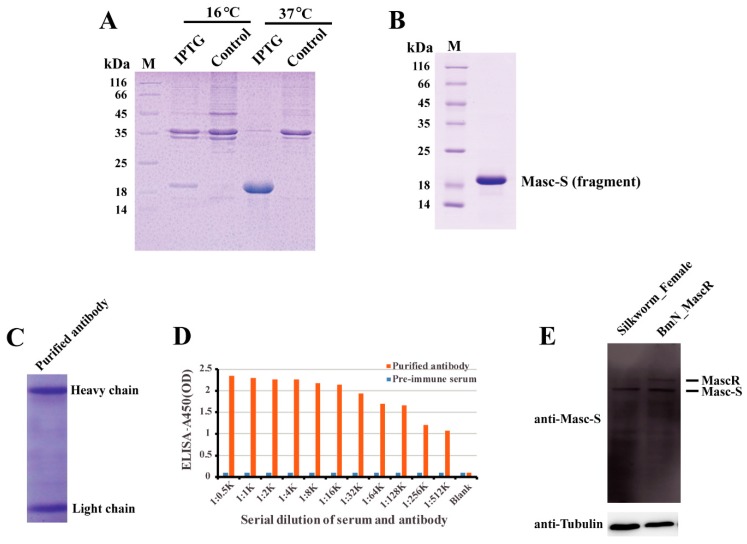
Preparation of polyclonal antibody and detection of the Masc-S protein in the female silkworm. (**A**) Analysis of the recombinant Masc-S (fragment) protein in *E. coli* Transetta(DE3) by SDS-PAGE. Lane M: Protein marker, lane IPTG: *E. coli* Transetta containing pET-28a-Masc-S(fragment)-His was induced with IPTG, lane control represents the control. (**B**) Purification of the recombinant Masc-S (fragment) protein. Lane M: Protein marker. (**C**) Analysis of purified polyclonal antibody of Masc-S by SDS-PAGE. (**D**) Titer analysis of Masc-S antibody by ELISA. The Masc-S antibody titer was more than 1:512000. Purified antibody starting dilution: 1:500. Optical density (OD) was detected via a microplate reader at 450 nm. (**E**) Detection of the Masc-S protein in the female silkworm and cell line by Western blot. Lane Silkworm_Female represents nucleoprotein extract from the female silkworm. lane BmN_MascR represents nucleoprotein extract from the cell line BmN in which MascR were over-expressed. α-Tubulin was used for the internal control.

In order to detect the Masc-S protein in the silkworm, we performed the preparation of polyclonal antibody against Masc-S. Firstly, we cloned the fragment of Masc-S CDS into the pET-28a vector, and prokaryotic expression showed that the recombinant Masc-S (fragment) protein was over-expressed at 37 ℃ by using IPTG (Figure 3A). The Masc-S (fragment) protein was purified and the purity was more than 95% (Figure 3B). The recombinant protein was further confirmed as Masc-S by MS. Then, the purified Masc-S protein was applied to prepare the polyclonal antibody. Results indicated that the titer of the Masc-S antibody was more than 1:512000 (Figure 3C,D). Finally, we performed Western blot assay to detect the Masc-S protein in the silkworm by the polyclonal antibody. Results showed that the Masc-S protein is present in the female silkworm (Figure 3E).

### 3.3. Identification of the Bound Protein to the Lacking Sequence of Masc-S

**Figure 4:**
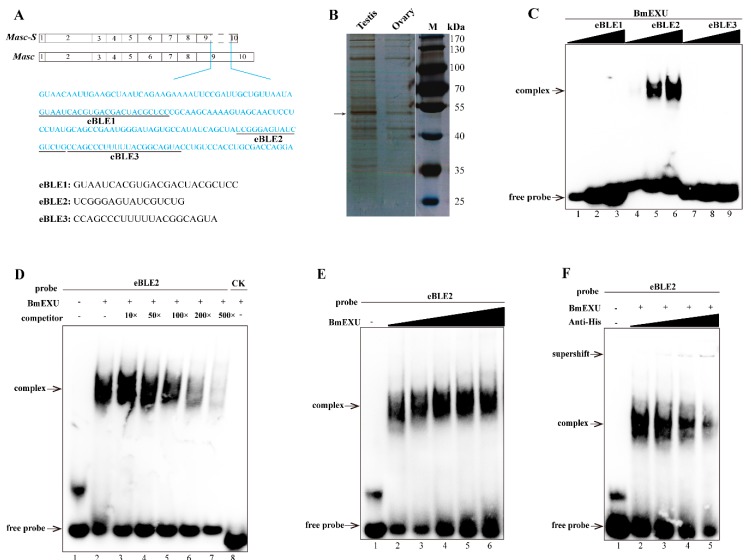
Identification of the protein specifically binding to the exclusive RNA sequence in *Masc* compared to *Masc-S*. (**A**) The exclusive RNA sequence (blue characters) in *Masc* was showed. In the exclusive sequence, there is three elements similar to the binding-like element 1 (BLE1) of *Drosophila bcd* gene, and we named three elements as eBLE1, eBLE2 and eBLE3. (**B**) SDS-PAGE with silver staining showed the proteins pulled down by the exclusive RNA from nucleoprotein extract from testis and ovary of day 5 fifth instar larvae. The different band (arrow) was excised from the gel for following LC/MS/MS analysis. MS/MS spectra were searched against the protein database, which resulted in the identification of 14 candidate binding proteins including *Bombyx mori* exuperantia (BmEXU) protein (Supplementary Table S2). In *Drosophila*, the EXU protein functions in the germline by binding the sequence of BLE1 in the *bcd* mRNA. (**C**) EMSA of the biotinylated probes (eBLE1, eBLE2 or eBLE3) and nucleoprotein extract from BmE with overexpression of the BmEXU protein was carried out. In order to verify whether the BmEXU protein binds to the exclusive RNA sequence in Masc compared to Masc-S, we performed EMSA of the BmEXU protein and three elements (eBLE1, eBLE2 or eBLE3) in the exclusive RNA sequence. The amount of RNAs (100, 200, 500 nmol) for each probe was incubated with nuclear extract (1.2 µg) for 20 min, respectively. (**D**–**F**) Gel-shift assay of the BmEXU protein and the eBLE2 RNA probe was performed to verify the specific binding of the protein to the probe. (**D**) The binding assays of BmEXU nuclear extract (1.2 µg) to the probe eBLE2 (150 nmol) with 1-, 10-, 50-, 100-, 200-, 500-fold excess cold probes (lanes 2–7) and an irrelevant probe as control check (lane 8: CK) were shown. (**E**) Different concentration of the BmEXU protein (lanes 1–6: 0, 0.6, 1.2, 1.8, 2.4, 3.6 µg) were incubated with eBLE2 (150 nmol). (**F**) Different concentration of anti-His-tag antibody (lanes 2–5: 0, 0.2, 0.5, 1.0 µL) incubated with eBLE2 (150 nmol) and BmEXU nuclear extract (1.2 µg). The recombinant His-tagged BmEXU protein was overexpressed in the BmE cells. The positions of the supershift, free and complexed RNAs are shown in arrows.

To address the issue of *Masc-S* generation, we employed the distinctive sequence between *Masc* and *Masc-S*, the lacking sequence of the latter, to perform an interactive protein down (Figure 4A). We performed SDS-PAGE to disperse the protein combined with the pretreated beads and stained with silver. A significant difference was found for a protein of approximately 52 kDa in the testis nuclear extract, as compared to bound proteins from male and female nuclear extracts (Figure 4B). Further, the result of LC/MS/MS revealed 14 candidate binding proteins (Supplementary Table S2). Interestingly, four unique peptides were detected, including residues 50 to 63, 127 to 139, 192 to 207, and 214 to 237, corresponding to the protein encoded by *B. mori exuperantia* (*Bmexu*). Previous studies showed that *Drosophila exu* protein functions in the germline and directs proper localization of the *bicoid* (*bcd*) mRNA by binding a preferred sequence of binding-like element 1 (BLE1) in the *bcd* 3’UTR during the earliest stages of oogenesis [21]. Based on the characteristics of the sequence of BLE1, rich in AU and AG, we searched for a similar element in the lacking sequence of *Masc-S*, and found three candidates, named as eBLE1, eBLE2, and eBLE3, which were designed correspondingly as Biotinylated RNA probes (Figure 4A). Subsequently, we constructed the over-expression vector of *Bmexu* (pSL1180-His-Bmexu) with His-tag to verify the relationship between BmEXU and eBLE1, 2, 3, by EMSA. Results suggested that the BmEXU protein could specifically bind to the eBLE2 probe in vitro (Figure 4C–F). Together, RNA-protein pull-down and EMSA indicated that BmEXU can specifically bind the probe eBLE2, which suggests that it has a possible role for BmEXU to bind different sections of *Masc-S* and full-length *Masc* to regulate alternative splicing of the *Masc* gene.

### 3.4. Roles of Masc-S in Genital Development

**Figure 5:**
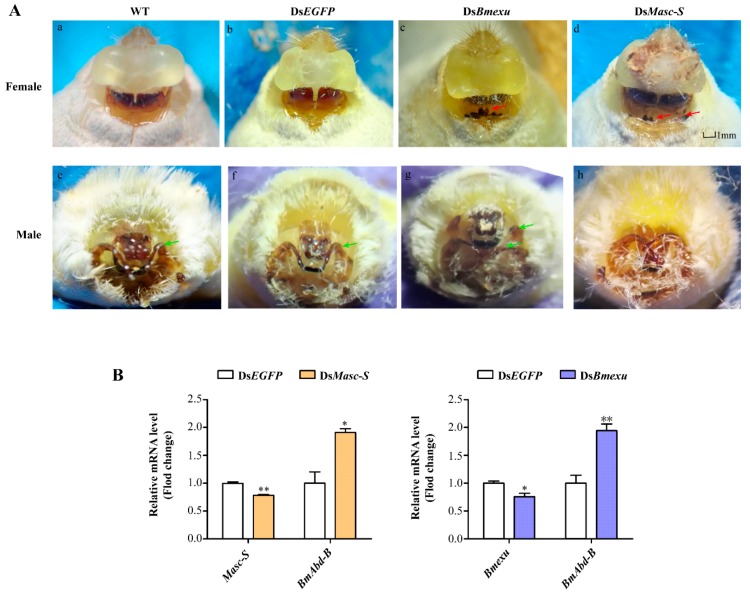
Effects of ds*Masc* and ds*Bmexu* injection on the female external genital morphology and detection of the expression of *Masc-S*, *Bmexu* and *Adb-B* after dsRNA injection. (**A**) WT represents wild type, and ds*EGFP* injection was performed as the control group. The red arrow means that black protuberances appeared in ventral plates. The green arrow represents clasper. (**B**) Expression levels of *Masc-S* and *Bmexu* were down-regulated using dsRNA of *Masc-S* and *Bmexu*, respectively. *Adb-B* was up-regulated in both two experiment groups. Different numbers of asterisks above the standard error bars indicate significant differences (* 0.01 < *p* < 0.05, ** *p* < 0.01).

To further study the function of *Masc-S*, we performed RNAi experiments in the silkworm. As remarked above, *Bmexu* may be involved in the generation of the *Masc-S* pattern, thus we carried out RNAi to down-regulate expression levels of *Masc-S* and *Bmexu*. The double-stranded RNA (dsRNA) was transcribed and synthesized in vitro, and *Masc-S* and *Bmexu* dsRNA were injected into female individuals on day 1 of the fifth-instar larvae according to the expression profile of *Masc-S* and *Bmexu* (Supplementary Figure S2). The morphology was observed in the moth phase, and downregulation of *Masc-S* induced the abnormal appearance of the external genitalia in the female (Figure 5A). In the ds*Masc-S* group, 30 female silkworms in total were injected by ds*Masc-S*, of which 14 silkworms produced an abnormal phenotype. The genital papillae showed a developmental disorder and the obvious black protuberances appeared in ventral plates (Figure 5A). In the dsBmexu group, 16 out of 30 female silkworms showed abnormal phenotype. By contrast, zero out of 30 silkworms were abnormal in the dsEGFP group. Compared with the group injected with ds*EGFP* dsRNA, the black protuberances caused by ds*Masc-S* RNAi are similar to that of the ds*Bmexu* group. Moreover, the total RNA was extracted from the ovary after dsRNA injection at 48 h for qPCR analysis. The RNAi knocking down efficiency of *Masc-S* and *Bmexu* genes seems not ideal. We also checked the mRNA levels of both two genes after 72-h injection, and the results is similar. A possible reason is that the high efficiency of RNAi on some genes in the silkworm is difficult to achieve [22,23]. However, we still found a significant reduction in the transcription levels of *Bmexu* and *Masc-S* after injection (Figure 5B). Further, we found that *Bmexu* or *Masc-S* downregulation could affect the mRNA level of other genes. *Abd-B* is required to specify the posterior abdomen and the genitalia in insects [16]. Interestingly, in female, for *Masc-S* RNAi, the expression level of *B. mori Abd-B* increased significantly, similar to the *Bmexu* RNAi group (Figure 5B).

We also performed RNAi of *Masc-S* and *Bmexu* in the male silkworm. Results showed that RNAi in the male led to a developmental disorder of the external genitalia in the ds*Masc-S* group and more one clasper in the ds*Bmexu* group (Figure 5A). Since Fem-derived piRNA could cleave the full-length *Masc* mRNA in the female, the functional full-length *Masc* is present in the male not in the female. In the female, *Masc-S* RNAi could target specifically to itself due to no functional full-length Masc. In the male, the *Masc-S* RNAi also targets to the full-length *Masc*, which probably led to no similar phenotype produced by RNAi of *Masc-S* and *Bmexu* in the male. 

**Figure 6:**
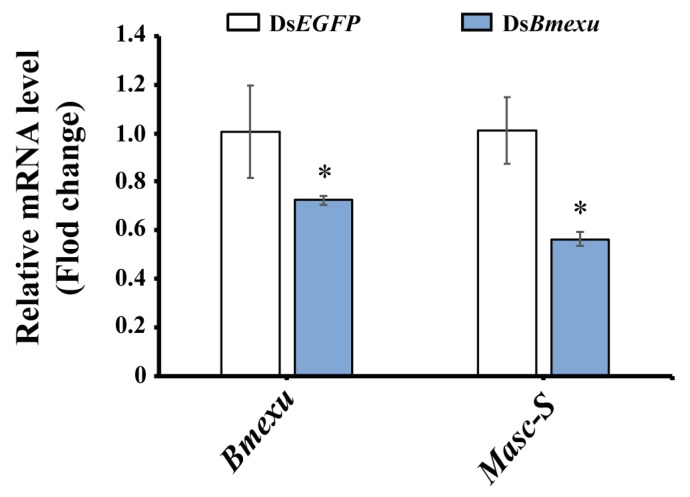
Expression level of *Masc-S* was down-regulated using dsRNA of *Bmexu* in the silkworm. The asterisk above the standard error bars indicates significant differences (* 0.01 < *p* < 0.05).

In order to address whether *Bmexu* regulates alternative splicing of the *Masc* gene, we detected the expression level of *Masc-S* after *Bmexu* RNAi. Results showed that *Masc-S* was down-regulated when the *Bmexu* gene was knocked down (Figure 6). Together with the binding of the BmEXU protein to the exclusive RNA sequence in *Masc* compared to *Masc-S*, we inferred that *Bmexu* regulated the splice variant *Masc-S* of the *Masc* gene. To summarize, our study indicated that a regulatory pathway in the female genital development is composed of *Bmexu*, *Masc-S* and *Abd-B*, and that *Bmexu* is the top, and *Adb-B* is in the bottom of the cascade.

## 4. Discussion

In the study, we identified a novel splicing isoform *Masc-S* differing from full-length *Masc* in its gene structure, and the exon 9 of *Masc-S* contained a *Fem* piRNA-cleavage-site defect. It was reported that the cleavage site in the ninth exon of *Masc* was bound by *Fem* piRNA through complementation, generating *Masc* piRNA [7]. Similarly, *Fem* was bound by *Masc* piRNA and spliced to generate *Fem* piRNA, resulting in the absence of full-length *Masc* protein in females. The sequence analysis of *Masc-S*, suggested that, compared with *Masc* piRNA, putative *Masc-S* piRNA may contain a mutation at the cleavage site of *Fem*, and it cannot generate *Fem* piRNA, thus expressing a functional protein by *Masc-S* which is antagonistic to the cleaving of *Fem* piRNA Futhermore, we found that the novel splicing isoform *Masc-S* was not regulated by *Fem* and could be translated into the protein production in the female.

Alternative splicing is a fundamental mechanism to modulate gene expression and regulate tissue and organ development [24], which causes the same pre-mRNA to produce a variety of splice variants, encoding many proteins with different roles from the full-length protein [25]. It is well-known that the phosphatidylinositol-3-kinase/protein kinase B (PI3K/Akt) signaling pathway is necessary to regulate cell proliferation, and its constitutive activation is related to tumor formation, invasion, and metastasis [26,27]. The Akt kinase family includes three subtypes: Akt1, Akt2, and Akt3. Akt3 generates two alternatively spliced variants, Akt3/-S472 and Akt3/+S472, the former lacks the key regulatory serine 472 phosphorylation site compared to the full-length (Akt3/+S472). While over-expression of Akt3/-S472 has been shown to suppress mammary tumorigenesis, Akt3/+S472 had no effect on malignant cell growth [28]. Thus, spliced variants have been demonstrated to perform different functions compared to the full-length protein. In our observation, the splicing isoform *Masc-S* can translate a protein without a frameshift mutation after alternative splicing. From the SMART website, the prediction showed that the Masc-S protein contained the same CCCH-type zinc finger domains as the full-length Masc. However, the C-terminal containing masculinization region of Masc-S was shorter, but retained two conserved sites, Cys-301 and Cys-304. RNAi for *Masc-S* down-regulated the expression of *Abd-B*, resulting in abnormal morphology in the exterior female genitalia. Our results suggested that *Masc-S*, due to alternative splicing events, participated in female external genital development, which is a different way from the masculinizing function of full-length *Masc*. 

Interestingly, BmEXU was identified to bind to the exclusive RNA sequence in *Masc* compared to *Masc-S* in this study. Previous studies have shown that *Drosophila* EXU initiates the correct localization of *bcd* in early embryo by binding BLE1 located on the *bcd* mRNA [29]. In 2016, the crystal structure of EXU was analyzed, which revealed that each monomer contained a 3’-5’ EXO-like domain and a sterile alpha motif domain, which could bind and modify the target RNA [30]. In *Drosophila*, *tra-2* regulates sex splicing of *dsx* and controls *exu* to produce male and female germline mRNAs with different untranslated regions [31]. In addition, x-ray mutagenesis screens were performed to generate flies with *exu* mutations, and analysis indicated that most mutations were sterile and had defects in spermatogenesis [32]. These results implied that the *Bmexu* gene probably functions in sex differentiation by regulating the splicing of the key sex-determining gene *Masc* in the silkworm. Furthermore, after performing RNAi for *Masc-S* and *Bmexu* in fifth-instar larvae, we obtained a similar effect of the abnormal genital phenotypes and expression of *Abd-B*. Thus, we suspect that *Bmexu* affects the transcription of *Masc* by regulating splicing, and *Masc-S* impacts morphological development in the female.

## 5. Conclusions

In conclusion, we identified a novel splicing variant *Masc-S* of the key sex-determining gene *Masc* and the regulatory factor BmEXU binding the lacking sequence of *Masc-S*. *Masc-S* was antagonistic to the cleaving of the piRNA derived from the sex-determination primary signal *Fem*, and played an important role in female external genital development. Our study provides clues for understanding the mechanism of sex determination and differentiation in the silkworm, which can be used as a reference for other lepidopterans.

## Supplementary Materials

The following are available online at https://www.mdpi.com/2218-273X/9/8/318/s1. Figure S1: PCR analysis of *Masc* transcripts in fifth instar larvae, Figure S2: Expression levels of Masc-S and Bmexu in the female during the fifth instar stage, Table S1: List of primer sequences used in this study, Table S2: Result of RNA-protein pull-down and LC-MS/MS analysis.
