# Supplementary material for: A Novel Splice Variant of the Masculinizing Gene Masc with piRNA-Cleavage-Site Defect Functions in Female External Genital Development in the Silkworm, Bombyx mori
Source: Biomolecules. 2019 Jul 30;9(8):318. doi: 10.3390/biom9080318 (PMC6723575; doi:10.3390/biom9080318)
Supplement: Supplementary file 1 [file biomolecules-09-00318-s001.pdf]

Supplemental Tables and Figures:

Table S1. List of primer sequences used in this study.

Primer name	Primer sequences (5'-3')	Purpose
Masc-f	ATGACATCGGCAAAAGTAGCAA	PCR
Masc-r	CTATTGAAACGGCGGTGGTGGT	PCR
Masc-S-r	TCACCGGTTGCTTTGAAGTCGA	PCR
E9-f	CTAGTAAAGAAAGAAAACCAG	PCR
CE910-f	AAAGAGGTATTGCTACAGCT	PCR
Bmdsx-f	AACCATGCCACCACTGATACCAAC	PCR
Bmdsx-r	GCACAACGAATACTGCTGCAATCG	PCR
seMasc-S-q-f	CAGCCGAATGGGATAGTGCC	qPCR
Masc-S-q-r	GGCGGTGGTGGTTGTGGATA	qPCR
Bmexu-q-f	GGTGAACAGCAAGAAGTAAGTG	qPCR
Bmexu-q-r	CGTGTTCTGTCTTTCAAGGATC	qPCR
SW22934-f	TTCGTACTGGCTCTTCTCGT	qPCR
SW22934-r	CAAAGTTGATAGCAATTCCCT	qPCR
Adb-B-f	CTATCCTCCAGATGCTCCCG3	qPCR
Adb-B-r	ACCCTGATGACAGCCTCCAT	qPCR
Masc-S-pro-f	CGGGATCCATGGACTGCAATAACTGTG	prokaryotic expression
Masc-S-pro-r	CCAAGCTTTCAACGCGCTTCTGGTT	prokaryotic expression
ovMasc-f	CGCGGATCCATGACATCGGCAAAAGTAGCAA	overexpression
ovMasc-r-Myc	TTGCGGCCGCCTACAGATCCTCTTCAGAGATGAGTTTCTGCTCTTGAAAC GGCGGTGGTGGT	overexpression
ovMasc-S-f	CGCGGATCCATGACATCGGCAAAAGTAGCAA	overexpression
ovMasc-S-r-Myc	ATAAGAATGCGGCCGCTCACAGATCCTCTTCAGAGATGAGTTTCTGCTCC CGGTTGCTTTGAAGTCGA	overexpression
ovBmexu-f′	CGGGATCCATGGCCATGGTGACTGAAGTGA	overexpression
ovBmexu-r-His	ATTTGCGGCCGCTTAATGGTGATGGTGATGATGGTTGGCCGCGATCGGCT CT	overexpression
eBLE-T7-f	TAATACGACTCACTATAGGGACTGCATTGATGAACAGCCCCG	Pull down
eBLE-r	AATCCTGGTCGCAGGTGGACAGG	Pull down
dsMasc-S-T7-f	TAATACGACTCACTATAGGCATATCGCGACTAACAGCG	RNAi
dsMasc-S-T7-r	TAATACGACTCACTATAGGATGCGATACTGGTATTGAG	RNAi
dsBmexu-T7-f	TAATACGACTCACTATAGGGGAAGCGAAATGGAAGCGAA	RNAi
dsBmexu-T7-r	TAATACGACTCACTATAGGGTAGCACACGAGCCAACACT	RNAi
dsEGFP-T7-f	TAATACGACTCACTATAGGACGTAAACGGCCACAAGTTC	RNAi
dsEGFP-T7-r	TAATACGACTCACTATAGGTGCTCAGGTAGTAGTGGTTGTCG	RNAi

Table S2. Result of RNA-protein pull down and LC-MS/MS analysis

Serial number	Matches	Score	Accession	Description
1	7	248	Q2F5T3	ATP synthase subunit alpha OS=Bombyx mori GN=LOC692926 PE=2 SV=1
2	4	243	Q2F6A0	Exuperantia OS=Bombyx mori PE=2 SV=1
3	4	77	Q1HPT0	ATP synthase subunit beta OS=Bombyx mori PE=2 SV=1
4	2	52	H9JEL8	Uncharacterized protein OS=Bombyx mori PE=4 SV=1
5	1	47	Q8T8B5	Tubulin alpha chain OS=Bombyx mori GN=bmtua2 PE=2 SV=1
6	1	40	H9JTB0	Uncharacterized protein OS=Bombyx mori GN=LOC101741115 PE=4 SV=1
7	1	34	Q0PZE7	eIF4AIII protein OS=Bombyx mori GN=eIF4AIII PE=2 SV=1
8	1	33	Q8T8B2	Tubulin beta chain OS=Bombyx mori GN=bmtub2 PE=2 SV=1
9	1	28	H9JJM6	Uncharacterized protein OS=Bombyx mori PE=4 SV=1
10	1	25	H9JAV9	Uncharacterized protein OS=Bombyx mori PE=4 SV=1
11	1	24	H9JBA1	Uncharacterized protein OS=Bombyx mori PE=4 SV=1
12	1	23	H9JTJ4	Uncharacterized protein OS=Bombyx mori PE=4 SV=1
13	1	19	H9J9X8	Uncharacterized protein OS=Bombyx mori GN=LOC101741342 PE=4 SV=1
14	1	14	H9JDR8	Elongator complex protein 3 OS=Bombyx mori PE=3 SV=1

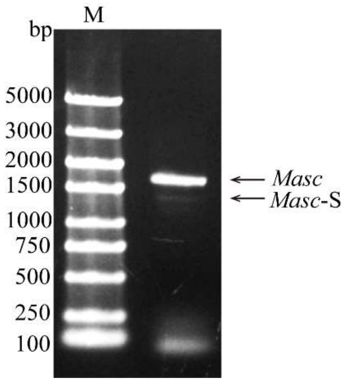

Figure S1. PCR analysis of *Masc* transcripts in fifth instar larvae. PCR was performed by the primers Masc-f and Masc-r (shown in Table S1), which overlapped full-length *Masc* CDS. The production of PCR was separated on a 1.2% agarose gel. Both two bands were cloned and sequenced.

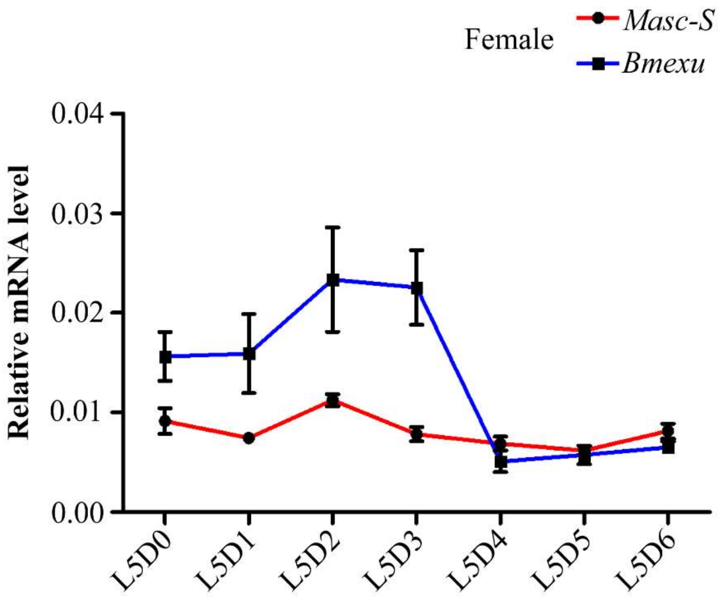

Figure S2. Expression levels of *Masc-S* and *Bmexu* in the female during the fifth instar stage. The qPCR was carried out by the primers Masc-S-q-f and Masc-S-q-r (for *Masc-S*), as well as Bmexu-q-f and Bmexu-q-r (for *Bmexu*). L5D0: molting before fifth instar; L5D1-L5D6: days 1-6 of fifth instar.
